# The Reliability of the Brief Visuospatial Memory Test - Revised in Brazilian multiple sclerosis patients

**DOI:** 10.1590/1980-57642018dn12-020014

**Published:** 2018

**Authors:** Marco Aurélio G. de Caneda, Daissy Liliana Mora Cuervo, Nathércia Estevam Marinho, Maria Cecília A. de Vecino

**Affiliations:** 1 Multiple Sclerosis Center, Department of Neurology, Hospital Moinhos de Vento, Porto Alegre, RS, Brazil.

**Keywords:** BVMT-R, BICAMS, cognitive impairment, multiple sclerosis, reliability, validity, BVMT-R, BICAMS, esclerose múltipla, cognição, validade, confiabilidade

## Abstract

Cognitive Impairment (CI) is a common and distressing problem in Multiple Sclerosis (MS). Its identification is complicated and sometimes omitted in the routine evaluation by neurologists. The BICAMS (Brief International Cognitive Assessment for Multiple Sclerosis) is a promising tool to overcome this difficulty. However, there is some concern regarding the subjectivity in scoring of the BVMT-R (Brief Visuospatial Memory Test - Revised), one of its components.

**Objective:**

To evaluate the reliability of the BVMT-R in a sample of Brazilian MS patients, with the measure being administered and scored by neurologists.

**Methods:**

BICAMS was applied to seventy subjects comprising forty patients diagnosed with MS and thirty healthy controls. In the MS patients group, the coefficients of agreement between three different raters, using the same protocols, and the internal consistency of the BVMT-R were assessed. Also, the coefficients of correlation of the BVMT-R with the other tests of the BICAMS, CVLT II (California Verbal Learning Test II) and SDMT (Symbol Digit Modalities Test), and their respective effect sizes were calculated.

**Results:**

the BVMT-R presented a moderate inter-rater coefficient of agreement (k=0.62), an excellent Intraclass Correlation Coefficient (ICC=0.85), and high internal consistency (α=0.92). The correlation between the BVMT-R and CVLT II was moderate (ρ=0.36; p<0.025), but strong with the SDMT (ρ=0.60; p<0.01), with a large effect size.

**Conclusion:**

The BVMT-R is a reliable instrument for assessing CI in patients with MS, having a significant association with information processing speed, an aspect which should be considered when evaluating its score.

## Resumo

O declínio cognitivo (DC) é um problema comum na esclerose múltipla (EM), mas sua identificação é complexa, por vezes sendo omitida na avaliação de rotina pelos neurologistas. O BICAMS é uma bateria breve de testes neuropsicológicos que visa superar tais dificuldades. No entanto, uma possível subjetividade no sistema de pontuação do BVMT-R, um dos seus componentes, é um ponto frágil desta ferramenta.

**Objetivo:**

Avaliar a confiabilidade do BVMT-R em pacientes portadores de EM, aplicado e aferido por neurologistas.

**Métodos:**

Os testes do BICAMS foram administrados a setenta indivíduos, quarenta pacientes portadores de EM e trinta controles saudáveis. No grupo de pacientes com EM foram calculados os coeficientes de concordância dos resultados entre três diferentes avaliadores e a consistência interna do BVMT-R. Também foram calculados os coeficientes de correlação do BVMT-R com os demais testes componentes do BICAMS e seus respectivos tamanhos de efeito.

**Resultados:**

O BVMT-R apresentou um coeficiente de concordância entre examinadores moderado (k=0,62), um excelente Coeficiente de Correlação Intraclasse (ICC=0,85) e uma alta consistência interna (α=0,92). A correlação entre o BVMT-R foi moderada (CVLT II: ρ=0,36; p<0,025) ou forte (SDMT: ρ=0,60; p<0,01), com grande tamanho de efeito.

**Conclusão:**

O BVMT-R é um instrumento confiável para avaliar o DC na EM, apresentando uma associação significativa com a velocidade de processamento da informação, o que deve ser considerado na interpretação de seus resultados.

Multiple sclerosis (MS) is the most common inflammatory disease of the Central Nervous System (CNS) among young adults of working age.^1^^,^ 2 MS can affect cognition very early,^3^^,^ 4 is usually progressive, and negatively impacts quality of life.^5^ The prevalence of Cognitive Impairment (CI) in MS ranges widely, from 40 to 70%,^3^^,^ 6 with similar rates found in Brazil.^7^^,^ 8

The most frequently affected cognitive domains include memory, information processing speed, visuospatial perception and attention.^4^ CI is one of the most important causal factors in poor adherence to treatment, unemployment, failures in rehabilitation, decrease in physical independence, social isolation, unsafe driving and changes in marital status of MS patients.^9^^-^ 11 CI is sometimes omitted in the routine evaluation of MS because the cognitive assessment still uses complex tests, demands a large series of sessions and requires a high investment.^12^ In addition, identifying CI may be difficult even for experts and some studies have demonstrated a low accuracy in its diagnosis.^13^

The undefined cut-off points and the large number of different Neuropsychological Tests (NPTs) used may preclude the uniformity of CI diagnosis.^14^ Patients with MS may have a metacognition deficit and do not recognize their own cognitive limitations, or confuse it with common problems in MS, such as depression or fatigue, and consequently do not report their complaints regarding CI in the routine evaluation. On the other hand, the cognitive changes of MS are usually more subtle than those found in other neurological conditions, such as the major neurocognitive disorders or vascular disease.^10^^,^ 13 ^,^ 14

The BICAMS (Brief International Cognitive Assessment for Multiple Sclerosis) is a short battery of NPTs that attempts to overcome these problems. It is a brief cognitive assessment tool, applicable in daily clinical practice, within the restricted time of a routine visit, with good sensitivity and specificity for screening and follow-up of CI in MS.^5^^,^ 6 ^,^ 9 ^,^ 15 The BICAMS can be used by any health professional, not requiring specialist training,^10^ and was recently validated in Brazil.^16^ The components of the BICAMS are: (1) the SDMT (Symbol Digit Modalities Test), which evaluates information processing speed and visual working memory, (2) the CVLT-II (California Verbal Learning Test II; only the first five recall trials, without the delayed trial), which is a verbal learning and memory test, and (3) the BVMT-R (Brief Visuospatial Memory Test - Revised; only the first three trials, without the delayed trial) to assess visuospatial learning and memory.^5^^,^ 9 ^,^ 15

Although the BICAMS is a promising tool, there is some criticism about its full utilization. The battery does not assess the executive functions, and the performance of subjects can be reduced by the physical disability caused by MS, low level of education, aging, use and/or abuse of psychoactive substances, and the presence of severe untreated anxiety or depression.^3^^,^ 9 Moreover, the presence of potential subjectivity in scoring of the BVMT-R, which does not occur in the other BICAMS tests, could lead to a variation between raters and generates a bias in determination of visuospatial memory impairment.^3^ The BVMT-R, whose psychometric properties were extensively studied and validated, including in Brazil,^17^ has a scoring system that may induce errors, with misinterpretation and inconsistencies in rating of location, rotation and preservation failures in the reconstructed images, which can affects the overall results.^3^^,^ 18

We explored the clinimetric and psychometric properties of the BVMT-R, particularly its reliability when used by people working daily in MS patient care, but without previous training on its application or the expertise of a specialist neuropsychologist.

## METHODS

### Participants

Forty (40) subjects with Relapsing-Remitting type MS, as defined by the McDonald Criteria 2010,^19^ forming the MS Group, and thirty (30) healthy controls selected from the local community, forming the HC Group, were included in this study. All subjects in both groups were older than 18 years and provided an informed consent form. The subjects in the MS Group were selected from the sample of a previously performed study, following approval of the local research ethics committee.^7^

Exclusion criteria were: (a) clinical conditions besides MS affecting CNS; (b) previously diagnosed cognitive disabilities secondary to conditions other than MS; (c) any prior impairment secondary to MS which precluded the application of the NPTs; (d) psychiatric illness, previous or developing, being treated or otherwise; (e) abuse of alcohol or other psychoactive substances; (f) MS attack treated with corticosteroids at high doses in the last six weeks; (g) Beck Depression Inventory ≥29 points, and/or Beck Anxiety Inventory ≥30 points performed by patients in HC group; and (h) Mini-Mental State Examination ≤25 points performed by patients in HC group older than 55 years.

### Evaluation tools and procedures

Patients with MS included were evaluated at regular visits and all NPTs of the BICAMS were applied individually by a senior neurologist (MAGC=E1). Matrices containing the drawings of the first three trials of the BVMT-R for each MS patient were later evaluated by two other researchers (DLMC=E2 and NSM=E3), resident doctors in Neurology program of Moinhos de Vento Hospital, Porto Alegre, RS, Brazil.

The application of the BICAMS and scoring of the BVMT-R were performed in accordance with instructions previously described in the literature,^5^^,^ 6 ^,^ 9 ^,^ 15 and the examiners did not undergo any previous practical training. The researchers provided their scores for the trials of the BVMT-R blinded to the clinical status of MS patients, patient performance on other NPTs of the BICAMS, and the scores provided by the other examiners. Only the E1 researcher applied the BICAMS to subjects in the HC group.

### Data analysis

The Shapiro-Wilk normality distribution test and a Dixon test to evaluate the presence of extreme values ​​(outliers) were performed for all the variables, which were provided by the raw scores of the NPTs. The differences between the mean scores of MS and HC Groups were evaluated by the Mann-Whitney test, t-Test or Chi-square test, as required. For the analysis of demographic data and clinical characteristics, the descriptive measures were expressed as N, means, standard deviations (sd) and percentages.

The Correlation Coefficients^20^ were calculated between: (1) the BVMT-R and SDMT of the MS Group, to check for a possible association between information processing speed and visuospatial learning memory; (2) the BVMT-R and CVLT II of the MS Group, checking the convergence validity; and (3) BVMT-R and Age, and BVMT-R and Level of Education of individuals in the MS Group. The confidence intervals (95% CI) of the Correlation Coefficients were calculated by the bootstrap resampling method, in the percentile mode. A regression analysis was performed between: (a) BVMT-R and SDMT, and (b) BVMT-R and CVLT II; controlling for Age and Level of Education. The Breusch-Pagan test was performed for homoscedasticity and the Durbin-Watson test assessed the presence of autocorrelation.

The Kappa (k) Concordance Coefficient^21^ was calculated to estimate the level of inter-rater agreement in ratings of the drawings (D1 to D6) for each of the first three trials (T1 to T3) of the BVMT-R performed by the MS patient group. We also calculated the Intraclass (ICC) Correlation Coefficient^20^ corresponding to scores of T1, T2 and T3, and to the overall scores of BVMT-R in the MS group. The internal consistency of the BVMT-R was evaluated by calculating the Cronbach Coefficient of Homogeneity (α).^22^ On the measurement of the Correlation Coefficients, only the scores by E1 were used, but in the calculation of K, ICC and α the scores provided by the E1, E2 and E3 examiners were used.

Finally, effect sizes were estimated: (1) η^2^ for the difference between the BVMT-R scores of MS and HC Groups; (2) Cohen’s q for the difference between the Correlation Coefficients MS group of BVMT-R × SDMT and BVMT-R × CVLT II; of BVMT-R × SDMT and BVMT-R × Age; and of BVMT-R × SDMT and BVMT-R × Education Level. Statistical significance was set at a value of p<.05 and statistical analyses were performed using Stata™14.1 Copyright^©^ 1985-2015, StataCorp LP, Statistics/Data Analysis StataCorp^®^, 4905 Lakeway Drive, College Station, Texas 77845 USA, available for free use at http://www.stata.com, accessed from June 26 to July 26, 2016.

## RESULTS

The Shapiro-Wilk test indicated a non-normal distribution of BVMT-R in both groups of subjects, and thus the Spearman coefficient (ρ) was used for the assessment of Correlation Coefficients. The Dixon test did not indicate the presence of significant outliers in the variables of the study. The demographic features and mean scores of the NPTs of the BICAMS are presented in Table 1.

**Table 1:** Demographic data of sample and BVMT-R results.

Group		Healthy control (n=30)		Multiple sclerosis (n=40)		p value
Age (years)*		40.03 (18-74)		42.67 (21 – 67)		.18^NS^
Education level*		(1) ^£^ n=4 (13.3%)		(1) n=6 (15%)		–
		(2)^&^ n=8 (26.6%)		(2) n=13(32.5%)		–
		(3) ^¶^ n=11(36.6%)		(3) n=14(35%)		–
		(4) ^¥^n=7 (23.3%)		(4) n=7 (17.5%)		–
Gender (F:M)**		8 (26.6%): 22 (73,3%)		11 (27.5%): 29 (72.5%)		.93^NS^
BVMT-R - Mean (Sd)*		26.3 (6.83)		22.57 (7.48)		.04
SDMT - Mean (Sd)***		56.7(15.39)		48.1(18.5)		.04
CVLT II - Mean (Sd)*		49.2(8.94)		48.65(8.71)		.26^NS^
EDSS - Mean (Sd)		–		3.44(1.28)		–

* Mann-Whitney Test;

** Chi-Square Test;

*** t-Test;

£ ≤8;

& > 8-11;

¶ > 11-18;

¥ > 18 years of education;

NS not significant.

The MS group showed a moderate and statistically significant correlation between the BVMT-R and CVLT II (ρ=0.36; 95% C.I.=0.05 to 0.59; p<0.025), and a strong, highly significant correlation between the BVMT-R and SDMT (ρ=0.60; 95% C.I.=0.35 to 0.76; p<0.01). We found no significant correlations between the BVMT-R and Age (ρ= -0.17; p>0.25) or between the BVMT-R and Education Level (ρ=0.05; p>0.70). The HC group also showed no significant results for these correlations. The BVMT-R identified CI, defined by 1.5 Sd below the mean scores of the HC group, in 22.5% of MS patients.

**Figure 1:**
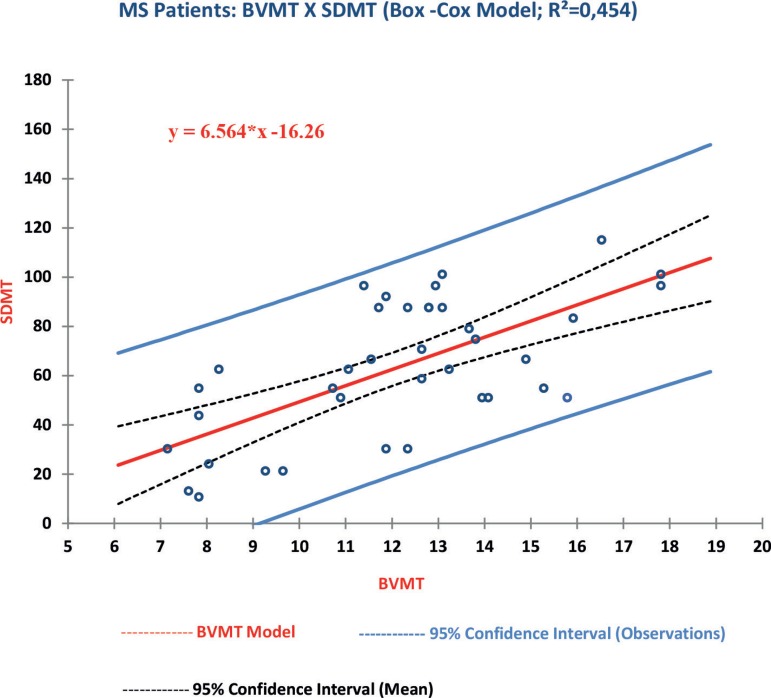
Regression BVMT-R × SDMT.

**Figure 2:**
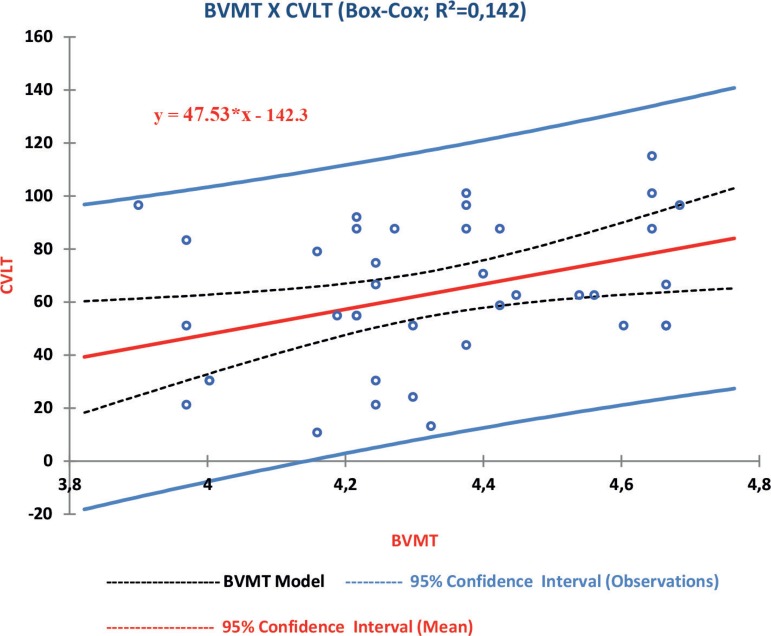
Regression BVMT-R × CVLT.

Autocorrelation was not detected among the analyzed variables, but there was heteroskedasticity in its residuals. In an attempt to correct this distortion, these variables underwent a Cox-Box transformation. After this procedure, a regression analysis between BVMT-R and SDMT showed that an increase of 2.5 points on the SDMT would generate an increase of 1 point on the BVMT-R (Figure 1), and around 45% of the variation of the BVMT-R could be attributable to variations in the SDMT score (p<0.05). The same analysis performed between the BVMT-R and CVLT II demonstrated that an increase of 3 points on the CVLT II would implicate an addition of 1 point on the BVMT-R (Figure 2), however, only 14% of the variation in BVMT-R scores could be explained by the CVLT II scores.

The k coefficient of agreement between raters of the first three trials of the BVMT-R had a mean value of 0.62 (Table 2), indicative of good clinically acceptable agreement. Only one figure, the D2 of T3, showed a weak agreement. This particular result was probably compromised by a common problem related to the Kappa Statistic, a distortion known as prevalence bias.^21^ The ICC indicated excellent agreement between the examiners for total scores of each trial and for overall BVMT-R scores (Table 3). The MS group had a very good a Cronbach coefficient of 0.92 (95% C.I.=0.89 to 0.94) and the HC group had a good coefficient of 0.75 (95% C.I.=0.66 to 0.83), both indicative of satisfactory reliability by the method of internal consistency.

**Table 2:** Kappa Coefficients of Trials T1 to T3 of Drawings D1 to D6 of BVMT-R.

Trial	Drawing						
	D1	D2	D3	D4	D5	D6	Mean
T1*	0.61 (0.48-0.75)	0.50 (0.37-0.64)	0.78 (0.65-0.91)	0.84 (0.68-1)	0.55 (0.42-0.69)	0.58 (0.43-0.73)	0.64 (0.50-0.78)
T2*	0.61 (0.47-0.75)	0.50 (0.36-0.64)	0.66 (0.52-0.88)	0.77 (0.63-0.90)	0.57 (0.44-0.70)	0.70 (0.57-0.83)	0.63 (0.49-0.78)
T3*	0.78 (0.61-0.95)	0.23 (0.06-0.41)**	0.65 (0.51-0.80)	0.75 (0.61-0.88)	0.66 (0.53-0.80)	0.58 (0.45-0.72)	0.60 (0.46-0.71)
Mean	0.66 (0.52-0.81)	0.41 (0.26-0.56)	0.69 (0.56-0.86)	0.78 (0.64-0.92)	0.59 (0.46-0.73)	0.62 (0.48-0.76)	0.62

* p<0.01;

** p<0.05.

**Table 3:** Intraclass Correlation Coefficients of BVMT-R.

Trial	Coefficient	
	ICC**	95% C.I.***
T1*	0.86	0.78-0.92
T2*	0.85	0.74-0.92
T3*	0.80	0.67-0.89
BVMT-R ^¶^	0.85	0.75-0.91

* Sum of the Trial;

** Intraclass Correlation Coefficient;

*** Confidence Interval;

¶ Total Score of test.

In order to calculate the effect size on the differences of BVMT-R averages between the MS and HC groups, we calculated η^2^, which was 0.059, representing a moderate effect size. Converting this rating to Cohen’s d effect size, the index would be 0.51 (95% C.I.=- 0.03 to 0.99), also indicating a moderate effect size, with a Cohen U3 index of 69.3, and a probability of superiority (probability of any component of the HC group having a greater score than any component of the MS group) of 64%. The Cohen’s q effect size (used for Correlation Coefficients) in the differences between the coefficients r of the correlation BVMT-R X SDMT compared to BVMT-R x Age was 0.86; and of the correlation BVMT-R x SDMT compared to BVMT-R x Education Level was 0.63, both indicative of a large index. The Cohen’s q effect size for the differences between the coefficient r of BVMT-R x CVLT II compared to the BVMT-R x Age was 0.55, and of BVMT-R x CVLT II compared to BVMT-R x Education Level, was 0.32, both considered median effects.

## DISCUSSION

The mean overall score of the BVMT-R in the MS group in this study was 22.5, similar to the value of 21.5 reported in the literature.^6^^,^ 16 ^,^ 18 ^,^ 23 ^-^ 35 The percentage of 22.5% of CI identified by the BVMT-R in our sample was lower than the average of 34% reported in previous studies.^6^^,^ 23 ^,^ 30 ^,^ 32 ^,^ 35 This lower rate is possibly due to a smaller total proportion of CI patients among the MS patients in our sample, of only 30%, when compared to the cited studies, in which the mean rate of CI was 56.5%.

As in several previous publications, this study showed a significant difference between the mean score of the BVMT-R obtained for the HC and MS groups, suggesting the criterion or discriminant validity of BVMT-R in this setting.^6^^,^ 23 ^,^ 34 ^,^ 35 The effect size of Cohen’s d=0.51 in this difference was extremely close to the mean index of 0.55 reported in the literature.^16^^,^ 29 ^,^ 31 ^,^ 34 The significant correlation BVMT-R x CVLT II may indicate the convergent validity of BVMT-R and the significant ρ coefficient of this correlation is consistent with previous publications, supporting the notion that the BVMT-R is a valid tool for assessing visuospatial memory, even when using only its learning trials.^16^^,^ 28 In general, as observed in our results, the BVMT-R validity data found previously are replicated in Brazil.

The strong association between the BVTM-R and the SDMT in our study points to an influence of information processing speed in the visuospatial memory. This finding is reinforced in the regression analysis between these variables, with a great impact of the SDMT scores on the variation of BVMT-R. This is a naturally expected result, as the BVMT-R learning trials are timed, and thus, good performance depends on processing speed. This impact did not occur in the regression analysis between the BVMT-R and Age or Level of Education, showing that the influence of these variables on the BVMT-R is much smaller than processing speed. Besides that, the higher effect sizes indicated by the Cohen q index in the Correlation Coefficients of the BVMT-R x SDMT compared to the coefficients of correlation of the BVMT-R x Age, and BVMT-R x Level of Education, reinforces the important association of processing information speed and the BVMT-R detected in regression analysis.

It would be possible to attribute part of the sensitivity of the BVMT-R in distinguishing MS patients from normal controls by the marked slowness of processing of information in these patients, since it is not uncommon for MS patients to experience slowed processing speed. Some authors consider the exposure time of 10 seconds to the matrix of figures of the BVMT-R too short, or the inclusion of six different figures for reproduction to be excessive.^36^ A bad performance on the BVMT-R may denote not only a visuospatial memory impairment, but also slower information processing speed. Perhaps the application of some correction ratios to BVMT-R results could offset the losses in information processing speed among MS patients.

Notably, the correlation between the BVMT-R and Age in the MS group was weak. This is in discordance with several previous publications in which this association was strong.^5^^,^ 16 ^,^ 23 ^,^ 25 ^,^ 28 ^,^ 33 However, in other studies this correlation was low, as found in the present study.^36^^,^ 37 Our negative result in this association again reinforces the influence of information processing speed on BVMT-R. Processing speed is affected by age, and our significant result in the correlation between the SDMT and Age confirms this association (ρ=- 0.39; p<0.02). Thus, the low impact of Age on the BVMT-R in our results suggests a specific relationship between information processing speed and the BVMT-R, regardless of patient age.

Akin to Age, the degree of formal education of the subjects in our sample exerted a very small influence on the BVMT-R score variation. There are previous studies in which this correlation was also not significant.^5^^,^ 35 A recent Canadian publication, which also showed a weak association between the BVMT-R and Level of Education, postulated that this result could be due to a higher educational level of the patients, since the correlation of education with other NPTs of the BICAMS showed the same negative results.^35^ However, in our study this causal effect cannot be argued, as the remaining NPTs of the BICAMS had highly significant Correlation Coefficients with Level of Education (SDMT: ρ=0.45; p<0.005 and CVLT II: ρ=0.34; p<0.05). Apparently the exact intensity of the correlation between the BVMT-R and Level of Education remains elusive, because other authors also describe a very significant association of these variables without the bias of a higher educational level of subjects.^25^^,^ 28

Determining the accuracy of the components of the BICAMS is essential to avoid misdiagnosis or omissions in the detection of CI in MS patients. For this reason we evaluated the concordance of scores of the BVMT-R obtained by different examiners, or inter-rater agreement, and its internal consistency, or homogeneity. Each patient in the MS group made a total of 18 attempts to reconstruct figures assessed by three raters, generating 2160 scores, 720 **K** coefficients of agreement and 160 ICCs. These numbers reinforce the robustness of the coefficients described in our study. Besides **K** coefficients higher than 0.60, the ICCs and the coefficient α, both greater than 0.80, indicate clinically satisfactory reliability of BVMT-R.^21^^,^ 27 Unfortunately, to our knowledge, there are no prior publications with this specific analysis to compare against our findings.

There are some methodological limitations of the present study that warrant comment. The sample size could raise questions about the external validity of some results. Possibly, the non-significant correlation between the BVMT-R and Age could have been the result of this supposed bias. Besides, the non-significant correlation of the BVMT-R and Level of Education may have resulted from an uneven distribution of the number of individuals in each category of this variable. Nevertheless, the reliability of NPTs in patients with MS seems to be robustly determined with samples as small as 20 individuals.^22^

In conclusion, despite its somewhat subjective scoring system, the BVMT-R seems to be a reliable instrument for assessment of visuospatial learning and memory, and CI detection and monitoring in MS patients, with adequate performance for clinical practice, even without specific dedicated or previous training in its application. Its psychometric properties include a significant association with information processing speed, and, for a more accurate evaluation, this should be considered in the assessment of MS patients.

## References

[1] Bohlega S (2014). Epidemiology of MS. Mult Scler Relat Disord.

[2] Browne P, Chandraratna D, Angood C, Tremlett H, Baker C, Taylor B, Thompson AJ (2014). Atlas of Multiple Sclerosis 2013: A Growing Global Problem with Widespread Inequity. Neurology.

[3] McNicholas N, McGuigan C (2016). A Useful Annual Review of Cognition in Relapsing MS is Beyond Most Neurologist - YES. Mult Scler J.

[4] Smestad C, Sandvik L, Landro NI, Celius EG (2010). Cognitive Impairment after Three Decades of Multiple Sclerosis. Eur J Neurol.

[5] Goretti B, Nicolai C, Hakiki B, Sturchio A, Falautano M, Minacapelli E (2014). The Brief International Cognitive Assessment for Multiple Sclerosis (BICAMS): Normative Assessment for Gender, Age and Education Corrections in the Italian Population. BMC Neurology.

[6] Dusankova JA, Kalincik T, Havrdova E, Benedict RHB (2012). Cross Cultural Validation of the Minimal Assessment of Cognitive Function in Multiple Sclerosis (MACFIMS) and the Brief International Cognitive Assessment for Multiple Sclerosis (BICAMS). Clin Neuropsychol.

[7] Caneda MAG de, Vecino MAC de (2016). The Correlation between EDSS and Cognitive Impairment in MS patients. Assessment of a Brazilian Population Using a BICAMS Version. Arq Neuropsiquiatr.

[8] Negreiros MA, Landeira-Fernandez J, Kirchmeyer CV, Paes RA, Alvarenga R, Mattos P (2010). Cognitive profile of Brazilian individuals with relapsing-remitting multiple sclerosis. J Bras Psiquiatr.

[9] Langdon D, Amato MP, Boringa J, Brochet B, Foley F, Fredrikson S (2012). Recommendations for a Brief International Cognitive Assessment for Multiple Sclerosis (BICAMS). Mult Scler J.

[10] Langdon D (2016). A Useful Annual Review of Cognition in Relapsing MS is Beyond Most Neurologist - NO. Mult Scler J.

[11] Patti F, Nicoletti A, Messina A, Bruno E, Fermo S Lo, Quattrocchi G (2015). Prevalence and Incidence of Cognitive Impairment in Multiple Sclerosis: a Population- Based survey in Catania, Sicily. J Neurol.

[12] Hutchinson M (2016). A Useful Annual Review of Cognition in Relapsing MS is Beyond Most Neurologist - Commentary. Mult Scler J.

[13] Romero K, Shammi P, Feinstein A (2015). Neurologists' Accuracy in Predicting Cognitive Impairment in Multiple Sclerosis. Mult Scler Relat Disord.

[14] Fischer M, Kunkel A, Bublak P, Hoffmann F, Sailer M, Faiss JH (2014). How Reliable is the Classification of Cognitive Impairment Across Different Criteria in Early and Late Stages of Multiple Sclerosis. J Neurol Sci.

[15] Benedict RHB, Amato MP, Boringa J, Brochet B, Foley F, Fredrikson S (2012). Brief International Cognitive Assessment for Multiple Sclerosis (BICAMS): International Standards for Validation. BMC Neurology.

[16] Spedo CT, Frndak S, Marques VD, Foss MP, Pereira DA, Carvalho LF (2015). Cross-cultural Adaptation, Reliability and Validity of the BICAMS in Brazil. Clin Neuropsychol.

[17] Miotto EC, Campanholo KR, Rodrigues MM, Serrao VT, Lucia MSC de, Scaff M (2012). Hopkins verbal learning test-revised and brief visuospatial memory test-revised: preliminary normative data for the Brazilian population. Arq Neuropsiquiatr.

[18] Gaines JJ, Gavett RA, Lynch JJ, Bakshi R, Benedict RHB (2012). New Error Type and Recall Consistency Indices for the Brief Visuospatial Memory Test Revised: Performance in Healthy Adults and Multiple Sclerosis Patients. Clin Neuropsychol.

[19] Polman CH, Reingold SC, Banwel B, Clanet M, Cohen JA, Filippi M (2011). Diagnostic Criteria for Multiple Sclerosis: 2010 Revisions to the McDonald Criteria. Ann Neurol.

[20] Streiner D, Norman G (1995). Health Measurements Scales: a Practical Guide to their Development and Use. Reliability.

[21] Altmann DG (1990). Practical Statistics for Medical Research.

[22] Hobart JC, Cano SJ, Warner TT, Thompson AJ (2012). "What Sample Sizes for Reliability and Validity Studies in Neurology?". J Neurol.

[23] Argento O, Smerbeck A, Pisani V, Magistrale G, Incerti CC, Caltagirone C (2016). Regression-based Norms for the Brief Visuospatial Memory Test Revised in Italian Population and Application in MS Patients. Clin Neuropsychol.

[24] Benedict RHB (2005). Effects of Using Same versus Alternate-form Memory Tests during Short-interval Repeated Assessments in Multiple Sclerosis. J Int Neuropsychol Soc.

[25] Giedraitiené N, Kizlaitiené R, Kaubrys G (2015). The BICAMS Battery for Assessment of Lithuanian-Speaking Multiple Sclerosis Patients: Relationship with Age, Education, Disease Disability, and Duration. Med Sci Monit.

[26] Goverover Y, Chiaravalloti N, DeLuca J (2015). Brief International Cognitive Assessment for Multiple Sclerosis (BICAMS) and performance of Everyday Life Tasks: Actual Reality. Mult Scler J.

[27] Hoogs M, Kaur S, Smerbeck A, Weinstock-Guttman B, Benedict RHB (2011). Cognition and Physical Disability in Predicting Health-Related Quality of Life in Multiple Sclerosis. Int J MS Care.

[28] Kane KD, Yochim BP (2014). Construct Validity and Extended Normative Data for Older Adults for the Brief Visuospatial Memory Test Revised. Am J Alzheimers Dis Other Demen.

[29] Niccolai C, Portaccio E, Goretti B, Hakiki B, Giannini M, Pastó L (2015). A Comparison of the Brief International Cognitive Assessment for Multiple Sclerosis and the Brief Repeatable Battery in Multiple Sclerosis Patients. BMC Neurology.

[30] O'Connell K, Langdom D, Tubridy N, Hutchinson M, McGuigan C (2015). A Preliminary Validation of the Brief International Cognitive Assessment for Multiple Sclerosis (BICAMS) tool in an Irish Population with Multiple Sclerosis. Mult Scler Relat Disord.

[31] Parmenter BA, Testa SM, Schretlen DJ, Weinstock-Guttman B, Benedict RHB (2010). The Utility of Regression-based Norms in Interpreting the Minimal Assessment of Cognitive Function in Multiple Sclerosis (MACFIMS). J Int Neuropsychol Soc.

[32] Sandi D, Rudisch T, Füvesi J, Fricska-Nagy Z, Huszka H, Biernacki T (2015). The Hungarian Validation of the Brief International Cognitive Assessment for Multiple Sclerosis (BICAMS) battery and the Correlation of Cognitive Impairment with Fatigue and Quality of Life. Mult Scler Relat Disord.

[33] Strober L, Englert J, Munschauer F, Weinstock-Guttman B, Rao S, Benedict RHB (2009). Sensitivity of Conventional Memory Tests in Multiple Sclerosis: Comparing the Rao Brief Repeatable Neuropsychological Battery and the Minimal Assessment of Cognitive Function in MS. Multiple Sclerosis.

[34] Vanotti S, Smerbeck A, Benedict RHB, Caceres F (2016). "A New Assessment Tool for Patients with Multiple Sclerosis from Spanish-speaking Countries: Validation of the Brief International Cognitive Assessment for Multiple Sclerosis (BICAMS) in Argentina". Clin Neuropsycho.

[35] Walker LAS, Osman L, Berard JA, Rees LM, Freedman MS, MacLean H (2016). Brief International Cognitive Assessment for Multiple Sclerosis (BICAMS): Canadian Contribution to the International Validation Project. J Neurol Sci.

[36] Tam JW, Schitter-Edgecombe M (2013). The Role of Processing Speed in the Brief Visuospatial Memory Test Revised. Clin Neuropsychol.

[37] Gale SD, Baxter L, Connor DJ, Herring A, Comer J (2007). Sex Differences on the Rey Auditory Verbal Learning Test and the Brief Visuospatial Memory Test Revised in the Elderly: Normative data in 172 participants. J Clin Exp Neuropsychol.

